# Job-related exhaustion risk variant in *UST* is associated with dementia and DNA methylation

**DOI:** 10.1038/s41598-024-62600-3

**Published:** 2024-06-13

**Authors:** Sonja Sulkava, Jari Haukka, Karri Kaivola, Fatma Doagu, Alexandra Lahtinen, Katri Kantojärvi, Kalle Pärn, Priit Palta, Liisa Myllykangas, Raimo Sulkava, Tiina Laatikainen, Pentti J. Tienari, Tiina Paunio

**Affiliations:** 1 Department of Public Health and Welfare, Finnish Institute for Health and Welfare, Helsinki, Finland; 2 Department of Psychiatry and SleepWell Research Program, Faculty of Medicine, Helsinki University Central Hospital, University of Helsinki , Helsinki, Finland; 3 Department of Clinical Genetics, Helsinki University Hospital, Helsinki, Finland; 4 Department of Public Health, University of Helsinki, Helsinki, Finland; 5 Translational Immunology Program, Department of Neurology, Brain Centre, Helsinki University Hospital, University of Helsinki, Helsinki, Finland; 6 Neuroscience Center, Helsinki Institute of Life Sciences, University of Helsinki, Helsinki, Finland; 7 Research Program in Systems Oncology, Research Programs Unit, Faculty of Medicine, University of Helsinki, Helsinki, Finland; 8 Institute for Molecular Medicine Finland (FIMM), HiLIFE, University of Helsinki, Helsinki, Finland; 9 Institute of Genomics, University of Tartu, Tartu, Estonia; 10 Department of Pathology, University of Helsinki and HUSLAB, Helsinki University Hospital, Helsinki, Finland; 11 Institute of Public Health and Clinical Nutrition, University of Eastern Finland, Kuopio, Finland; 12 Amia Memory Clinics, Helsinki, Finland; 13 Joint Municipal Authority for North Karelia Social and Health Services (Siun Sote), Joensuu, Finland

**Keywords:** Genetic association study, Dementia, DNA methylation, Alzheimer's disease

## Abstract

Previous genome-wide association and replication study for job-related exhaustion indicated a risk variant, rs13219957 in the *UST* gene. Epidemiological studies suggest connection of stress-related conditions and dementia risk. Therefore, we first studied association of rs13219957 and register-based incident dementia using survival models in the Finnish National FINRISK study surveys (N = 26,693). The AA genotype of rs13219957 was significantly associated with 40% increased risk of all-cause dementia. Then we analysed the *UST* locus association with brain pathology in the Vantaa 85+ cohort and found association with tau pathology (Braak stage) but not with amyloid pathology. Finally, in the functional analyses, rs13219957 showed a highly significant association with two DNA methylation sites of *UST*, and *UST* expression. Thus, the results suggest a common risk variant for a stress-related condition and dementia. Mechanisms to mediate the connection may include differential DNA methylation and transcriptional regulation of *UST*.

## Introduction

Psychiatric disorders and symptoms have been studied as risk factors for dementia and Alzheimer’s disease (AD) in recent years, and there is growing evidence supporting the idea that individuals’ lifetime stress and mental health can affect future dementia risk.

Experiences of psychological stress, exhaustion, as well as stressful life events have been linked to increased risk of dementia in several studies^1–3^. Some studies have focused specifically on work-related stress and reported mostly significant associations with dementia^4–8^. Of the stress-related traits, the most robust support is for individual proneness to stress as a risk factor for AD^9–13^. The individual component of stress reactions is also likely to include genetic risk factors.

Heritability for job-related exhaustion has been estimated to be about 30%^14,15^. To our knowledge, thus far the only genome-wide studies on genetic risk factors for job-related exhaustion or burnout have been done by us^16,17^, and these studies discovered and replicated risk variants in *UST,* and specifically among shift workers, in *MTNR1A*. The *MTNR1A* variant was also associated with dementia, clinical AD and the neuropathological changes of AD in the very elderly^18^. Here, to further examine the potential genetic connection of job-related exhaustion and dementia, we focused on the association of the *UST* variant rs13219957 with incident dementia and AD in a large Finnish population-based FINRISK Study and with the neuropathology of AD in a very elderly Finnish cohort, Vantaa 85+.

## Results

### Cross-sectional associations of rs13219957 in the FINRISK study

This study included 26 693 individuals. The baseline characteristics and incident dementia and mortality according to rs13219957 genotype are shown in Table 1. In the cross-sectional data, the self-reported work-related stress scores were higher in carriers of the minor allele A of rs13219957 (Pearson Chi-Square test *P* = 0.019) (Table 1), similarly to what has been reported previously for the FINRISK Study surveys 1997–2002^16^. In addition, carriers of the A allele reported more insomnia symptoms (Pearson Chi-Square test *P* = 0.001). Non-AD dementia was more prevalent in carriers of the A allele of rs13219957. Otherwise, no significant differences between the genotype groups were present for the study traits and covariates when examining the data cross-sectionally. Individuals with missing genotype data (9.2%) were, on average, older and predominantly men, with a greater amount of cardiovascular risk factors and exhaustion symptoms. They more often encountered outcomes of dementia or death, yet the age at which dementia onset occurred was not significantly earlier (Supplementary Table S1).

**Table 1:** Baseline characteristics and incident dementia and mortality of the study population according to rs13219957 genotype.

Trait	Number of individuals with data for the trait	All rs13219957 genotypes	Rs13219957 GG (% in the genotype group or SD for quantitative traits)	Rs13219957 GA (% in the genotype group or SD for quantitative traits)	Rs13219957 AA (% in the genotype group or SD for quantitative traits)	Test of difference P value
Female sex, (%)	26,693	14,254 (53.4)	10,376 (53.2)	3568 (53.7)	310 (56.2)	0.34^b^
Baseline age, mean (SD)	26,693	47.7 (13.1)	47.7 (13.1)	47.8 (13.2)	47.8 (13.2)	0.83^a^
Age at the censoring (censored only), mean (SD), y	22,021	63.0 (12.4)	63.0 (12.4)	63.0 (12.3)	62.7 (12.4)	0.81^ s^
All-cause dementia, (%)	26,693	1540 (5.8)	1118 (5.7)	378 (5.7)	44 (8.0)	0.08^b^
Age of onset for all-cause dementia, mean (SD), y	1540	76.1 (7.5)	76.1 (7.6)	76.2 (7.2)	74.9 (9.0)	0.57^a^
AD, (%)	26,693	1257 (4.7)	919 (4.7)	308 (4.6)	30 (5.4)	0.69^b^
Age of onset for AD, mean (SD), y	1257	79.1 (7.0)	76.5 (6.7)	76.6 (6.8)	76.9 (5.5)	0.92^a^
Non-AD dementia. (%)	26,693	283 (1.1)	199 (1.1)	70 (1.1)	14 (2.7)	0.0025^b^
Age of onset for non-AD dementia, mean (SD), y	283	74.3 (10.1)	74.4 (10.5)	74.4 (8.6)	70.7 (13.2)	0.41^a^
Deaths, (%)	26,693	3824 (14.3)	2764 (14.2)	982 (14.8)	78 (14.1)	0.48^b^
Death age, mean (SD), y	3824	71.5 (11.2)	71.5 (11.1)	71.4 (11.3)	73.3 (10.5)	0.37^a^
Death age all-cause dementia, mean (SD), y	692	79.9 (6.5)	80.0 (6.7)	79.8 (6.1)	78.5 (7.8)	0.54^a^
Death age AD, mean (SD), y	500	80.6 (6.0)	80.5 (6.2)	80.8 (5.4)	80.1 (5.7)	0.83^a^
Death age non-AD dementia, mean (SD), y	192	78.1 (7.6)	78.6 (7.6)	77.2 (7.1)	75.9 (10.4)	0.34^a^
Occupational traits						
Years of education, mean (SD)	26,379	11.9 (3.9)	11.9 (3.9)	11.9 (4.0)	12.0 (4.0)	0.82^a^
Work-related stress, (%)	15,067	2506 (16.6)	1814 (16.4)	624 (16.8)	68 (22.4)	0.019^b^
Insomnia often/sometimes, (%)	26,320	2233/10,068 (8.5/38.3)	1582/7327 (8.2/38.1)	590/2510 (9.0/38.3)	61/231 (11.2/42.4)	0.002^b^
Cardiovascular risk factors						
Total cholesterol, mean (SD), mmol/L	26,680	5.5 (1.1)	5.5(1.1)	5.5 (1.1)	5.4 (1.0)	0.25^a^
BMI, mean (SD), kg/m^2^	26,622	26.7 (4.7)	26.7 (4.7)	26.7 (4.7)	26.7 (5.1)	0.93^a^
Systolic blood pressure, mean (SD), mmHG	26,682	134.8 (19.7)	134.9 (19.6)	134.8 (19.9)	134.0 (19.6)	0.57^a^
Self-reported diabetes, %)	24,785	854 (3.4)	615 (3.4)	221 (3.6)	18 (3.5)	0.82^b^
Physical activity low, (%)	25,313	2095 (8.3)	1512 (8.2)	538 (8.5)	45 (8.5)	0.14^b^
Physical activity moderate, (%)	25,313	6705 (36.0)	6705 (36.3)	2198 (34.8)	205 (38.8)	0.14^b^
Physical activity high, (%)	25,313	14,110 (55.7)	10,247 (55.5)	3584 (56.7)	279 (52.7)	0.14^b^
Smoking current/ex, (%)	26,502	6545/58,322 (4.7/22.0)	4784/4267 (24.7/22.0)	1628/1449 (24.7/22.0)	116/113 (21.2/24.3)	0.98^b^

*SD* standard deviation, *AD* Alzheimer’s disease, *BMI* body mass index.

^a^One-way ANOVA, equal variances and means.

^b^Pearson Chi-Square test.

### Associations of rs13219957 with incident dementia and competing risk of death in the FINRISK study

Association of rs13219957 genotypes with the risk of all-cause dementia and AD was studied using two survival models in parallel, accounting for the competing risk of death, the Poisson cause-specific hazard model and Fine-Gray sub-distribution hazard model. We refer here to the fully adjusted models if not otherwise stated. The AA genotype of rs13219957 showed association with an increased risk of all-cause dementia in both Poisson model (IRR = 1.40(1.03–1.94), and Fine-Gray model (HR = 1.55(1.13–2.13)). Association of AA genotype of rs13219957 with AD was not significant in either model (Poisson model, IRR = 1.18(0.80–1.73), Fine-Gray model, HR = 1.26(0.86–1.85)). The association was strongest for the cases of all-cause dementia without a diagnosis of AD separately (Poisson model, IRR = 2.27(1.25–4.12), Fine-Gray model, HR = 2.64(1.46–4.77)), but here cases of dementia with AA genotype numbered only 12. These results are presented in Table 2.

**Table 2:** Association of rs13219957 with incident all-cause dementia, Alzheimer’s disease (AD), non-AD dementia, and the competing risk of death.

Trait	N basic Model (cases)	N fully adjusted model (cases)	Poisson cause-specific hazard model		Fine-Gray sub-distribution hazard model	
			Basic model* IRR (CI 95%)	Fully adjusted model** IRR (CI 95%)	Basic model*** HR (CI 95%)	Fully adjusted model**** HR (CI 95%)
All-cause dementia rs13219957						
GG	19,223 (1062)	17,017 (897)	1.00	1.00	1.00	1.00
GA	6565 (362)	5841 (310)	0.99 (0.88–1.12)	1.01 (0.89–1.15)	0.98 (0.87–1.10)	0.99 (0.87–1.13)
AA	546 (42)	495 (39)	1.36 (0.99–1.85)	1.40 (1.03–1.94)	1.44 (1.06–1.96)	1.55 (1.13–2.13)
Alzheimer’s disease rs13219957						
GG	19,223 (877)	17,017 (746)	1.00	1.00	1.00	1.00
GA	6565 (294)	5841 (250)	0.98 (0.85–1.11)	0.98 (0.85–1.13)	0.96 (0.84–1.10)	0.96 (0.83–1.11)
AA	546 (29)	495 (27)	1.14 (0.79–1.65)	1.18 (0.80–1.73)	1.18 (0.82–1.71)	1.26 (0.86–1.85)
Non-AD dementia rs13219957						
GG	19,223 (185)	17,017 (151)	1.00	1.00	1.00	1.00
GA	6565 (68)	5841 (60)	1.08 (0.82–1.44)	1.19 (0.88–1.60)	1.07 (0.81–1.41)	1.15 (0.85–1.55)
AA	546 (13)	495 (12)	2.29 (1.31–4.02)	2.27 (1.25–4.12)	2.46 (1.40–4.32)	2.64 (1.46–4.77)
Competing risk of death rs13219957						
GG	19,223 (2196)	17,017 (1926)	1.00	1.00	1.00	1.00
GA	6565 (778)	5841 (688)	1.05 (0.99–1.12)	1.06 (1.00–1.13)	1.04 (0.96–1.13)	1.04 (0.95–1.13)
AA	546 (52)	495 (45)	0.81 (0.67–0.99)	0.71 (0.58–0.88)	0.79 (0.60–1.03)	0.69 (0.50–0.93)

*Model adjusted for FINRISK survey year, sex, educational class.

**Model adjusted for FINRISK survey year, sex, educational class, body mass index, systolic blood pressure, total cholesterol, smoking, physical activity, and diabetes. Follow-up time and age at the end of the follow-up were accounted for in the Lexis data frame.

***Model adjusted for sex and educational class.

****Model adjusted for sex, educational class, body mass index, systolic blood pressure, total cholesterol, smoking, physical activity, and diabetes. *AD* alzheimer’s disease, *IRR* incidence rate ratio.

**Figure 1:**
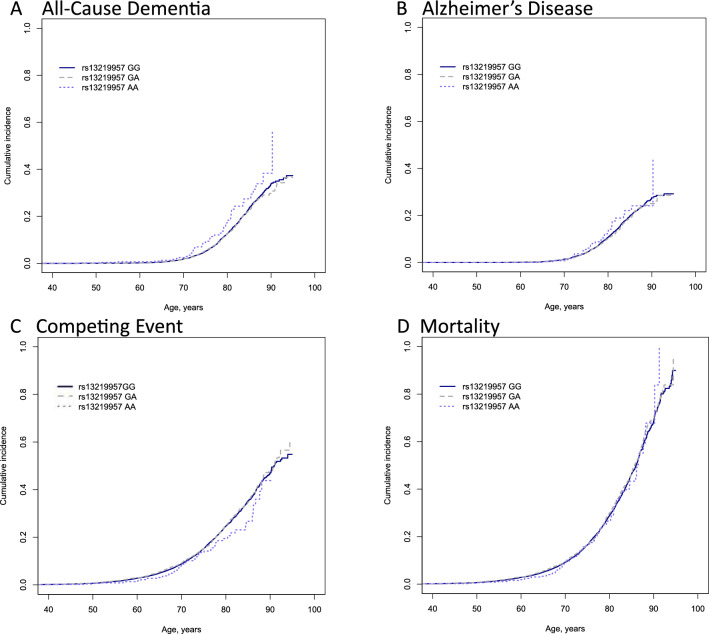
Cumulative incidence of all-cause dementia (**A**), Alzheimer’s disease (**B**), competing event, death without dementia (**C**), and mortality (**D**) by rs13219957 genotypes in the FINRISK Study surveys.

Rs13219957 AA also showed a significant association with a decreased risk of competing event, death without dementia, (Poisson model, IRR = 0.71(0.58–0.88); Fine-Gray model, HR = 0.69(0.50–0.93), Table 2). When analysing rs13219957 AA against all-cause mortality, no significant association was detected (Poisson model, IRR = 0.81(0.63–1.06)). Figure 1 demonstrates the cumulative incidence patterns for the different genotype groups. The cumulative incidence of all-cause dementia as well as AD are increased in the carriers of the AA genotype after 70 years of age, and the incidence of the competing event is lower after 55 years of age, and more clearly after 75 years. No difference is seen in the all-cause mortality between the genotype groups until the age of 90 years.

### Secondary analyses in the FINRISK study

In the secondary analyses, correction for the population components C1–C3 from the GWAS did not change the results when compared to the uncorrected analysis, suggesting that population stratification did not affect the results, but in the smaller sample for the GWAS data, the association of rs13219957 AA was non-significant (Supplementary Table S2).

To examine the impact of missing genotype data on our main results, analyses using the basic Poisson model for rs13219957, as shown in Table 2, were conducted again incorporating a separate category for missing genotype data. The effect sizes obtained were very similar to those in Table 2. Only the association between AA genotype of rs13219957 and all-cause dementia now included 1.00 within the confidence intervals, IRR = 1.37(1.00–1.86) (Supplementary Table S3). The rs13219957 genotypes were in accordance with Hardy–Weinberg Equilibrium (HWE) (Supplementary Table S4).

Next, we examined whether the association of rs13219957 AA with all-cause dementia was mediated by the experience of work-related stress, insomnia, or depressive mood. Covariation for insomnia did not affect the strength of the association of rs13219957 AA with dementia (Poisson model, IRR = 1.39(1.01–1.91)) (Supplementary Table 5). Covariation for work-related stress and depressive mood strengthened the association (Poisson model, IRR = 1.80(1.08–2.98) and IRR = 1.47 (1.06–2.05), respectively) in the sample that included the FINRISK Study surveys 1992–2002 but not 2007 in which the questions were unavailable (Supplementary Table S5). Work-related stress and the AA genotype of rs13219957 did not show statistically significant interaction (interaction terms: Poisson model, IRR = 2.09(0.69–6.34)).

### Fine mapping of the *UST* locus

We performed variant fine mapping of the genetic loci in the FINRISK Study surveys. By considering genetic linkage disequilibrium around rs13219957, we observed a haplotype block of 16 kb (bp 148,936,606–148,953,102; rs10457837–rs2500512). The minor allele A of rs13219957 (allele frequency 0.147) was included in only one haplotype, while the major allele G occurred on nine different haplotypes. In the haploblock area of rs13219957, 18 SNPs showed r^2^ > 0.2 with rs13219957. Of those variants, 10 demonstrated significant associations with all-cause dementia in the Poisson model using the GWAS data of the FINRISK Study (Table 3). The effect size was the highest for the original SNP rs13219957.

**Table 3:** Associations of the rs13219957-linked variants (r^2^ > 0.2 in the same haploblock area) with incident all-cause dementia and Alzheimer’s disease in the fully adjusted Poisson cause-specific hazard model.

SNP	All-cause dementia		Alzheimer’s disease	
	N (cases)	Poisson cause-specific hazard model fully adjusted* HR (CI 95%)	N (cases)	Poisson cause-specific hazard model fully-adjusted* HR (CI 95%)
rs10457837				
TT	15,235 (863)	1.0	15 235 (718)	1.0
TA	5458 (317)	1.03 (0.91–1.17)	5458 (255)	0.99(0.86–1.15)
AA	492 (35)	1.31 (0.93–1.84)	492 (24)	1.07(0.72–1.62)
rs6570912				
GG	8094 (433)	1.0	8094 (364)	1.0
GC	10,111 (587)	1.04 (0.92–1.18)	10,111 (479)	1.01(0.88–1.16)
CC	3036 (198)	**1.23 (1.04–1.45)**	3036 (156)	1.15(0.95–1.39)
rs1017851				
CC	6343 (334)	1.0	6343 (278)	1.0
CT	10,504 (623)	1.08 (0.94–1.23)	10,504 (513)	1.07(0.92–1.23)
TT	4360 (257)	1.09 (0.93–1.28)	4360 (205)	1.05(0.87–1.25)
rs992811				
CC	8066 (431)	1.0	8066 (362)	1.0
CG	10,109 (587)	1.04 (0.92–1.18)	10,109 (479)	1.01(0.88–1.16)
GG	3050 (198)	**1.23 (1.04–1.45)**	3050 (156)	1.15(0.95–1.39)
rs979429				
TT	6339 (334)	1.0	6339 (279)	1.0
TC	10,529 (624)	1.08 (0.94–1.23)	10,529 (513)	1.06(0.91–1.22)
CC	4390 (260)	1.09 (0.93–1.28)	4390 (207)	1.04(0.87–1.25)
rs3734375				
GG	6361 (336)	1.0	6361 (281)	1.0
GA	10,250 (601)	1.07 (0.93–1.22)	10,250 (490)	1.04(0.90–1.20)
AA	4139 (242)	1.18 (0.92–1.28)	4139 (195)	1.05(0.87–1.26)
rs9322161				
GG	8091 (432)	1.0	8091 (364)	1.0
GT	10,079 (587)	1.05 (0.93–1.19)	10,079 (478)	1.01(0.88–1.16)
TT	3009 (194)	**1.22 (1.03–1.44)**	3009 (153)	1.14(0.94–1.37)
rs6936210				
TT	8080 (431)	1.0	8080 (362)	1.0
TC	10,103 (586)	1.04 (0.92–1.18)	10,103 (478)	1.01(0.88–1.16)
CC	3032 (196)	**1.22 (1.03–1.45)**	3032 (155)	1.15(0.95–1.39)
rs7769776				
AA	7329 (388)	1.0	7329 (326)	1.0
AG	10,251 (594)	1.05 (0.93–1.20)	10,251 (488)	1.03(0.90–1.19)
GG	3412 (222)	**1.25 (1.06–1.47)**	3412 (174)	1.16(0.97–1.40)
rs1482393				
CC	7322 (386)	1.0	7322 (324)	1.0
CT	10,350 (599)	1.06 (0.93–1.20)	10,350 (491)	1.04(0.90–1.19)
TT	3491 (227)	**1.25 (1.06–1.47)**	3491 (179)	1.17(0.98–1.41)
rs12193341				
CC	15,261 (864)	1.0	15 261 (719)	1.0
CT	5446 (317)	1.03 (0.91–1.17)	5446 (255)	0.99(0.86–1.15)
TT	495 (34)	1.27 (0.90–1.79)	495 (24)	1.08(0.72–1.62)
rs6570913				
GG	8099 (433)	1.0	8099 (364)	1.0
GA	10,122 (588)	1.04 (0.92–1.18)	10,122 (479)	1.01(0.88–1.16)
AA	3037 (197)	**1.22 (1.03–1.45)**	3037 (156)	1.15(0.96–1.39)
rs6941730				
CC	8096 (433)	1.0	8096 (364)	1.0
CT	10,120 (588)	1.04 (0.92–1.18)	10,120 (479)	1.01(0.88–1.16)
TT	3037 (197)	**1.22 (1.03–1.45)**	3037 (156)	1.15(0.96–1.39)
rs11155608				
GG	8094 (433)	1.0	8094 (364)	1.0
GA	10,118 (588)	1.04 (0.92–1.18)	10,118 (479)	1.01(0.88–1.16)
AA	3033 (197)	**1.23 (1.04–1.45)**	3033 (156)	1.16(0.96–1.40)
rs10872625				
TT	7688 (415)	1.0	7688 (349)	1.0
TA	10,152 (590)	1.02 (0.90–1.16)	10,152 (484)	1.00(0.87–1.15)
AA	3293 (208)	1.18 (0.997–1.39)	3293 (161)	1.09(0.90–1.31)
rs10872626				
GG	8088 (433)	1.0	8088 (364)	1.0
GA	10,108 (588)	1.04 (0.92–1.18)	10,108 (479)	1.01(0.88–1.16)
AA	3032 (196)	**1.22 (1.03–1.44)**	3032 (155)	1.15(0.95–1.38)
rs1156561				
TT	7714 (416)	1.0	7714 (350)	1.0
TC	10,143 (592)	1.02 (0.90–1.16)	10,143 (485)	0.99(0.87–1.14)
CC	3283 (206)	1.16 (0.98–1.37)	3283 (160)	1.07(0.89–1.29)
rs2170534				
CC	6337 (332)	1.0	6337 (277)	1.0
CT	10,473 (624)	1.08 (0.95–1.24)	10,473 (512)	1.06(0.92–1.23)
TT	4352 (257)	1.09 (0.92–1.28)	4352 (206)	1.05(0.87–1.25)

*Model adjusted for FINRISK survey year, sex, educational class, body mass index, systolic blood pressure, total cholesterol, smoking, physical activity, and diabetes. Follow-up time and age at the end of the follow-up were accounted for in the Lexis data frame. Bold values represent significant associations at the CI 95% level.

### *UST* locus and post-mortem neuropathology in the Vantaa 85+ sample

**Figure 2:**
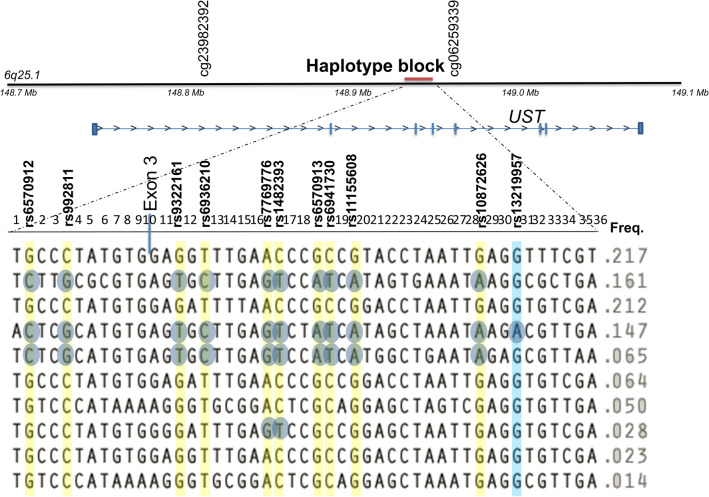
Genetic map for the *UST* gene region and rs13219957 haploblock. The haplotype block around rs13219957 is located at bp 148,936,606–148,953,102 (hg38) in the intron of *UST*. The haploblock SNPs in LD (r^2^ > 0.2) with rs13219957 and significant association with all-cause dementia in the FINRISK Study surveys and with BRAAK stage in the Vantaa 85+ are coloured yellow. The risk alleles of the SNPs are marked with blue circles. The two DNA methylation sites showing significant associations with the marked SNPs are shown in the top of the figure. Other SNPs of the haplotype block: 1 = rs10457837, 2 = rs1017851, 3 = rs17080218, 4 = 979,429, 5 = rs62426127, 6 = 62,426,128, 7 = rs9399677, 8 = rs6917705, 9 = rs9377180, 10 = rs3734375, 11 = rs9322160, 12 = rs449392, 13 = rs6941568, 14 = rs17080323, 15 = rs2449393, 16 = rs9373582, 17 = rs9390641, 18 = rs12193341, 19 = rs9390643, 20 = rs4895776, 21 = rs7747823, 22 = rs6570914, 23 = rs62426129, 24 = rs62426130, 25 = rs78603586, 26 = rs17728703, 27 = rs10872625, 28 = rs149809941, 29 = rs170080433, 30 = rs74860997, 31 = rs1156561 32 = rs2500511, 33 = rs12211515, 34 = rs2170534, 35 = rs12528390, 36 = rs2500512.

The 10 dementia-associated SNPs of the *UST* locus and rs13219957 were analysed for association with AD neuropathology. All the SNPs, except rs13219957, showed nominally significant (*P* < 0.01) association with the BRAAK staging score assessing the spread of neurofibrillary tangle tau pathology. No association was found with beta-amyloid load, clinical AD, or all-cause dementia (all *P* > 0.5). The results in the Vantaa 85 + cohort are presented in Table 4 and in Supplementary Figs. S1–S3. The risk alleles of the SNPs showing association with the BRAAK stage highlight three primary risk haplotypes, as shown in Fig. 2.

**Table 4:** Association of the SNPs in linkage disequilibrium with rs13219957 with neuropathology of Alzheimer’s disease and clinical dementias in Vantaa 85+.

Variant	BP	A1	R2 with rs13219957	MAF	BRAAK (N = 272)		CERAD (N = 272)		All-cause dementia (N_cases_ = 282, N_controls_ = 220)		Clinical Alzheimer’s disease N_cases_ = 133, N_controls_ = 220)	
					Beta (SE)	*P*	Beta (SE)	*P*	OR (CI 95%)	*P*	OR (CI 95%)	*P*
rs6570912	148,937,354	C	0.27	0.38	0.4 (0.13)	0.003	0.05 (0.09)	0.57	0.88 (0.67–1.15)	0.34	1.08 (0.79–1.48)	0.64
rs992811	148,937,579	G	0.27	0.38	0.4 (0.13)	0.003	0.05 (0.09)	0.57	0.88 (0.67–1.15)	0.34	1.08 (0.79–1.48)	0.64
rs9322161	148,942,806	T	0.27	0.38	0.4 (0.13)	0.003	0.05 (0.09)	0.57	0.88 (0.67–1.15)	0.34	1.08 (0.79–1.48)	0.64
rs6936210	148,943,189	C	0.27	0.38	0.4 (0.13)	0.003	0.05 (0.09)	0.57	0.88 (0.67–1.15)	0.34	1.08 (0.79–1.48)	0.64
rs7769776	148,944,435	G	0.23	0.41	0.4 (0.13)	0.006	0.02 (0.09)	0.79	0.95 (0.73–1.24)	0.69	1.18 (0.86–1.63)	0.31
rs1482393	148,944,810	T	0.24	0.41	0.37 (0.13)	0.006	0.02 (0.09)	0.79	0.95 (0.73–1.24)	0.69	1.18 (0.86–1.63	0.31
rs6570913	148,945,662	A	0.27	0.38	0.4 (0.13)	0.003	0.05 (0.09)	0.57	0.88 (0.67–1.15)	0.34	1.08 (0.79–1.48)	0.64
rs6941730	148,945,740	T	0.27	0.38	0.4 (0.13)	0.003	0.05 (0.09)	0.57	0.88 (0.67–1.15)	0.34	1.08 (0.79–1.48)	0.64
rs11155608	148,946,283	A	0.27	0.38	0.4 (0.13)	0.003	0.05 (0.09)	0.57	0.88 (0.67–1.15)	0.34	1.08 (0.79–1.48)	0.64
rs10872626	148,949,062	A	0.27	0.38	0.4 (0.13)	0.003	0.05 (0.09)	0.57	0.88 (0.67–1.15)	0.34	1.08 (0.79–1.48)	0.64
rs13219957	148,949,693	A	1	0.14	0.25 (0.17)	0.141	− 0.05 (0.11)	0.66	0.99 (0.70–1.42)	0.98	1.11 (0.73–1.69)	0.62

Linear regression analyses with sex as a covariate performed for measures of tau pathology (BRAAK stage) and beta-amyloid pathology (CERAD score), and logistic regression analyses with sex as a covariate for the diagnoses of dementia and Alzheimer’s disease.

*SNP* single nucleotide polymorphism, *OR* odds ratio, *BP* base pair, *A1* minor allele, *R2* square correlation measure for linkage disequilibrium, *MAF* minor allele frequency, *CI* confidence interval, *CERAD* consortium to establish a registry for Alzheimer’s disease.

### Association with DNA methylation, *UST* brain expression, and open chromatin region

To examine the potential functional mechanisms behind the genetic associations, we studied association of rs13219957 and the 10 other SNPs (marked in Fig. 2) with DNA methylation levels in the *UST* gene. DNA methylation was analysed in peripheral blood leukocytes in 454 individuals with non-missing covariates from the FINRISK Study survey of 2007. The basic characteristics of individuals for whom methylation data were either available or unavailable are reported in Supplementary Table S6. Associations of the 11 SNPs were calculated for all the available 92 CpG methylation sites in the *UST* gene area and UTRs. The rs13219957 minor allele showed a highly significant association with lower methylation levels at cg23982392 (P_FDR_ = 1.1 × 10^–8^) when multiple testing was corrected for and a less significant association with higher levels of cg062593339 (P_FDR_ = 0.012). The 10 other SNPs also showed association with lower methylation at cg23982392, but weaker than that of rs13219957 (Table 5).

**Table 5:** Significant associations of rs13219957 and the neuropathology-linked SNPs with DNA methylation levels in the *UST* gene.

Variant	cg23982392		cg06259339	
	P-value nominal/FDR corrected	Estimate	P-value nominal/FDR corrected	Estimate
rs6570912	9.62E−06/0.00089	− 0.22	NS	− 0.03
rs992811	1.84E−05/0.0017	− 0.21	NS	− 0.03
rs9322161	7.80E−06/0.00072	− 0.22	NS	− 0.03
rs6936210	9.62E−06/0.00089	− 0.22	NS	− 0.03
rs7769776	1.33E−05/0.0012	− 0.21	NS	− 0.03
rs1482393	7.72E−06/0.00071	− 0.22	NS	− 0.03
rs6570913	9.62E−06/0.00089	− 0.22	NS	− 0.03
rs6941730	9.62E−06/0.00089	− 0.22	NS	− 0.03
rs11155608	9.62E−06/0.00089	− 0.22	NS	− 0.03
rs10872626	9.62E−06/0.00089	− 0.22	NS	− 0.03
rs13219957	1.40e−9/1.29e−07	− 0.40	0.000193/0.008	− 0.08

Linear regression model was performed with sex, age, smoking status, alcohol consumption, array, slide and cell populations as covariates for 92 CpG sites and the SNPs (N = 454). Cg23982392, cg06259339 are situated in the body of *UST.*

*UST* uronyl-2-sulfotransferase gene, *FDR* false discovery rate, *NS* non-significant, *SNP* single nucleotide polymorphism.

The CpG site with the strongest association with the SNPs, cg23982392, is situated on top of a SNP rs75776863, which changes cytosine to thymine (T). The rare minor allele T of rs75776863, with frequency of 0.046, destroys the CpG site for DNA methylation. Our dataset includes two subjects with homozygosity for rs75776863 TT. Both had very low methylation M-values as supposed, and heterozygotes for rs75776863 had average methylation M-values (Supplementary Fig. S4). When analyzing LD, rs75776863 and rs13219957 showed R^2^ 0.081 and D’ 0.54. The TT homozygotes for rs75776863 were heterozygotes for rs13219957. Supplementary Fig. S4 demonstrates the LD between rs13219957 and rs75776863 and connection with cg23982392 methylation status.

The survival analyses for all-cause dementia using the FINRISK data were performed post hoc for rs75776863 heterozygotes (Supplementary Table S7). The analyses showed significant association with all-cause dementia in the basic model (IRR = 1.22(1.03–1.44)) but not in the fully adjusted model (1.17 (0.98–1.41)). The survival analyses were not possible for the TT homozygotes (N = 52) who were all dementia-free. When excluding TT homozygotes and heterozygotes for rs75776863, the strength of association of rs13219957 with all-cause dementia was not weakened suggesting also an independent effect of rs13219957 (Supplementary Table S8 vs. Supplementary Table S2).

The brain expression quantitative trait loci (eQTL) analyses using publicly available datasets revealed significant associations (*P* < 0.05) of the *UST* locus SNPs, excluding rs13219957, with lower *UST* brain expression in the ROSMAP^19^ and GTEx project data^20^. Borderline significant associations (*P* = 0.052) with the same direction of effect were observed in the AD patients but not in the non-AD patients in the eGWAS Mayo dataset^21^. No significant eQTL associations were detected in the microglial cell data^22^. Nevertheless, in microglial cells, the risk alleles of the *UST* locus SNPs (apart from rs13219957) demonstrated borderline significant negative associations (*P* = 0.032–0.055) with the open chromatin region (OCR) in the *UST* promoter region. The eQTL findings are presented in Supplementary Tables S9 and S10, and the OCR results in Supplementary Table S11.

## Discussion

This study revealed an association of rs13219957, a previously reported genetic risk variant for job-related exhaustion, with all-cause dementia in the large Finnish epidemiological FINRISK Study. No significant association with clinically defined AD was found, but association of the genetic locus with tau pathology of AD was detected in the smaller Vantaa 85+ cohort (though not with rs13219957). The strong association of rs13219957 with DNA methylation sites of the *UST* gene suggests epigenetic regulation of *UST* as a potential mechanism.

Previously, *UST* has not been listed as a risk gene for dementia or AD pathology. In contrast to the large multi-population studies, our Finns-only study may have identified specific genetic variants. Genetic homogeneity results in a reduced number of risk alleles (less allelic heterogeneity) and higher LD with longer haplotype blocks^23^. On the other hand, the use of survival models modelling competing risk of death is likely to increase our power when compared with multi-national cross-sectional analysis, and some weaker genetic signals may emerge. Summary statistics from the large GWAS for AD and AD-proxy^24^ show a weak but nominally significant association of rs13219957 with AD (*P* = 0.041, OR = 1.01(1.00–1.01), n = 452,010). That result, together with the association of the haplotype block with tau pathology, suggest that rs13219957 may also be a weak risk factor for AD and not only for all-cause dementia.

The strongest association signal in our study was observed, however, from the non-AD dementia cases. The mean age of onset of the dementia was 63.9 years, which indicates that among these are the early-onset cases (Table 1). Based on the prevalence of different dementias at this age, this non-AD group may consist of AD without adequate diagnostics or atypical symptoms, cases of dementia with Lewy bodies, vascular dementia, frontotemporal dementia, and alcohol-related dementia^25^. Tauopathies and hippocampal sclerosis are so rare that these are unlikely to contribute that signal, but unfortunately more specific diagnostics was not possible in our cohort.

Rs13219957 was significantly associated with methylation levels at two methylation sites, The association of the risk allele of rs13219957 with decreased methylation of cg23982392 is likely to be explained by weak LD with rare rs75776863, which can directly regulate possibility for methylation at that CpG site. However, our post hoc analyses suggested that rs75776863 would not explain the association of rs13219957 with dementia (Supplementary Table S4).

cg23982392 is situated only 26 bp from the binding site of CCCTC-binding factor (CTCF) in the intron of *UST*. Methylation in the CTCF binding site effectively inhibits binding of this multi-functional protein^26^, which functions for example as an insulator, chromatin remodeller, transcription factor and architectural protein^27^. Intronic CTCFs have been shown to control gene expression and regulate alternative splicing. Association of the *UST* locus SNPs with lower *UST* brain expression was, indeed, seen in two open datasets (Supplementary Table S8) suggesting that the association with dementia may be mediated by differential *UST* expression. Similar to the findings from the neuropathological analyses, the association signal was detected for other SNPs but not rs13219957. In line with the direction of the effect in the expression analyses, negative association with OCR in the promoter region of *UST* was seen for some of the *UST* locus SNPs (Supplementary Table S10). Interestingly, the same study highlighted *UST* as a gene with most significant association with cognitive resilience, lower *UST* expression correlating with greater cognitive decline.

UST functions as an enzyme adding sulfate groups to the uronyl residues of dermatan sulfate and, more weakly, to iduronyl residues of chondroitin sulfate^28^. Dermatan sulfate and chondroitin sulfate are intermediately sulfated glycosaminoglycans. In vitro, dermatan sulfate increases AD-like tau changes by stimulating tau phosphorylation, which is an early event in the formation of neurofibrillary tangles. The amount of sulfate groups in glycosaminoglycans correlates with higher amount of tau changes^29^. However, most of the evidence comes from the heparin sulfate^30^. We discovered association of the *UST* locus (except rs13219957) with Braak stage (Table 4) suggesting that this locus has an effect on tau pathology. However, the association of the *UST* locus risk alleles with lower *UST* expression conflicts with previous functional evidence suggesting that a higher amount of sulfation is linked to a higher amount of tau pathology. Thus, the molecular mechanisms behind these associations remain to be clarified.

Since mortality due to other causes is a competing event for dementia, we demonstrated here, in parallel, two survival models accounting for competing risk of death, Poisson and Fine-Gray, as recommended in^31^. Sometimes differences can be detected between aetiological risk factors, emphasised by the cause-specific hazard model, and factors that affect real cumulative incidence in the presence of competing events, emphasised by the Fine-Gray sub-distribution hazard model. In our study, associations of rs13219957 with all-cause dementia were significant in both fully adjusted models, Fine-Gray showing a slightly stronger association (Table 2).

In addition to all-cause dementia, we found association of rs13219957 with the competing risk of death (Table 2). Since no association of rs13219957 with all-cause mortality (which included cases of dementia) was detected, it is not likely to have a causal relationship with mortality, but the detected association is likely to be biased and caused by the competing effect of dementia diagnoses in our models^32^.

Individual differences in proneness to stress, assessed by the temperament trait neuroticism, have been linked to the risk of AD in several studies^9,12,13^, and no studies without significant association have been published to our knowledge. Previously, only our previous study of an *MTNR1A* variant has suggested genetic connection between proneness to stress (job-related exhaustion in shift work) and AD^17,18^. Here, the analyses adjusted for work-related stress suggested, however, that the association of rs13219957 is not mediated by increased susceptibility to experiencing work stress (Supplementary Table S4), but rs13219957 may show a separate risk effect for both conditions. Further studies, using for example polygenic risk scores, are needed to examine potential shared genetic risk factors for job-related exhaustion and dementia on a genome-wide scale.

This study has some limitations. First of all, the DNA methylation analyses were performed in blood leukocytes, while the more relevant cells for dementia and AD would be cortical or hippocampal neurons and glia.

Another limitation is the lack of disease diagnoses other than AD, which prevented studying the association of rs13219957 with specific diagnoses of non-AD dementia. We anticipated that the Finnish healthcare registers did not provide reliable enough clinical diagnoses for the most common types of dementia diseases after AD—vascular dementia and dementia with Lewy bodies—because no specific medications are available for either dementia type. For AD, the data on medication and special reimbursement was the most important source of diagnoses^1^. In addition, the clinical differentiation of different types of dementia (including AD) is challenging because of the commonness of the mixed pathologies for AD and vascular dementia and AD and dementia with Lewy bodies^33^.

Thirdly, the findings of this study cannot be generalized to the Finnish population due to selective participation overall^34^ and specifically in this genetic study (Supplementary Table S1). However, consistent results in the analyses that include the missing rs13219957 genotype as a separate category, along with genotype frequencies following HWE (Supplementary Table S4), suggest that the missing genotype data is unlikely to influence our results.

This large prospective study with careful statistical modelling suggests that a previously reported genetic risk variant for job-related exhaustion in middle-age, rs13219957 in *UST,* was associated with the risk of all-cause dementia. The association may be mediated by differential DNA methylation, transcriptional regulation of *UST,* and induction of tau pathology of AD. Association of rs13219957 with different types of dementia requires further studies.

## Method

### Participants

#### The national FINRISK study

The National FINRISK Study has examined the health and risk factors of the Finnish population. New participants were recruited every five years from 1972 to 2012^34^. Study subjects for this genetic study were derived from the National FINRISK Study surveys of 1992, 1997, 2002 and 2007. At baseline, the participants were 25–74 years old. For our analyses, the separate surveys were pooled together. Characteristics of our data by genotype groups are presented in Table 1. The study characteristics by survey are presented previously^34^.

The ethical committee of the Finnish Institute of Health and Welfare and/or the Coordinating Ethical Committee of Helsinki and Uusimaa Hospital District approved the study protocols for the FINRISK surveys 1992–2007.

#### Vantaa 85+ study

The Vantaa 85+ is a Finnish population-based cohort of the oldest old consisting of the over 85-year-old population of the Finnish city of Vantaa in 1991. All 553 participants were followed up until death with clinical examinations or via healthcare records. Of those, 534 gave their sample for the genetic study^18^. Of the Vantaa 85+ cohort, 304 individuals underwent post-mortem examination for neuropathology of AD^35^. The maximum density of beta-amyloid plaques was assessed with a CERAD (Consortium to Establish a Registry for Alzheimer’s disease) score^36^, and the spread of neurofibrillary tangles using BRAAK staging^37^.

The study protocols obtained permissions from the Local ethical committees. The Ministry of Social Affairs and Health granted permission to use the health and social care notes for the Vantaa 85+ study participants and the Center for Medico-Legal Affairs to collect and use autopsy tissue. All participants, and their relatives in case of dementia, gave their informed consent.

### Follow-up and events

The individuals were followed up from baseline to the first event, whichever occurred first: (1) diagnosis of dementia, (2) death without dementia, and (3) censoring at the end of the follow-up (end of 2017). Individuals with prevalent dementia were excluded (n = 26). Thus, depending on the FINRISK Study survey (1992, 1997, 2002 or 2007) the length of the follow-up was 10, 15, 20 or 25 years, respectively.

The diagnoses of dementia and AD were derived from the Finnish national registers which were linked to the FINRISK Study: the Causes of Death Register, the Hospital Discharge Register and the Drug Reimbursement Register and information from prescribed and purchased dementia drugs from the Social Insurance Institution. Information from the registers was combined for this study. The diagnosis of AD included, in 1992–1995, code 3310 ICD-9 (International Classification of Diseases) and from 1996 onwards, F00 and G30 (ICD-10). All-cause dementia was diagnosed if the individual fulfilled the criteria for AD or if they had codes 3310, 4378A, 290 (ICD-9) or from 1996 onwards F00, F01, F02, F03, G30 (ICD-10). In the Drug Reimbursement Register, AD was diagnosed based on the reimbursement for AD medication (Social Insurance Institution code 307 for ATC class N06D, anti-dementia drugs: donepezil, rivastigmine, memantine and galantamine). Cases of all-cause dementia but without AD (non-AD dementia) who did not die during the follow-up were censored in the analyses of AD. The definition of the events in the FINRISK Study is described in detail previously^1^.

### Genotyping

#### Genotyping in the FINRISK study

In the FINRISK Study surveys, genotyping for rs13219957 were performed using iPLEX Gold chemistry (Sequenom, San Diego, CA, USA) as described previously^16^. After quality control (QC), the genotype was available for 26,693 individuals. Genotypes for rs13219957 and the rs13219957-linked variants were also derived from GWAS genotyping. The genotyping was performed with Illumina beadchips (Human610-Quad, HumanOmniExpress, HumanCoreExome) at the Wellcome Trust Sanger Institute (Cambridge, UK), at the Broad Institute of Harvard and MIT (Boston, MA, USA), and at the Institute for Molecular Medicine Finland (FIMM) Genotyping Unit (Helsinki, Finland). Imputation was performed with SHAPEIT2 [23269371] and IMPUTEv2 [19543373] using a custom haplotype set of 2000 whole-genome-sequenced (WGS) Finnish individuals and 1000 Genomes project phase-3 haplotypes as reference panels. After QC for rs13219957, the number of individuals with genotypes was 24,028. Since, in the HumanCoreExome chip, rs13219957 was not directly genotyped but the genotype was estimated by imputation, we performed our main analyses with the iPLEX genotypes, which also offered a larger sample size, and used GWAS genotypes for fine mapping of the haplotype block area. Of the 22,869 individuals interrogated with different genotyping methods, 57 (0.25%) received different genotypes with the different methods. Principal component analysis (PCA) for population stratification was calculated with PLINK 1.9. The top three principal components (C1–C3) were used to adjust the effect estimates for population stratification.

#### Genotyping in Vantaa 85+ study

In the Vantaa 85+, genotyping was performed with Illumina Infinium Human370 BeadChips (Illumina, San Diego, CA, USA) as previously described^38^. Chip genotyped samples were merged with a larger dataset originating from a similar genotyping approach and pre-phased with Eagle 2.3.5 (https://data.broadinstitute.org/alkesgroup/Eagle/) with the default parameters, except the number of conditioning haplotypes was set to 20,000.

Genotype imputation was carried out by using the population-specific SISu v3 imputation reference panel with Beagle 4.1 (version 27Jan18.7e1, https://faculty.washington.edu/browning/beagle/b4_1.html) as described in the following protocol: 10.17504/protocols.io.nmndc5e. High-coverage (25–30×) WGS data used to develop the SISu v3 reference panel were generated at the Broad Institute of Harvard and MIT and at the McDonnell Genome Institute at Washington University, and jointly processed at the Broad Institute. The variant callset was produced with the GATK HaplotypeCaller algorithm by following GATK best-practices for variant calling. Genotype-, sample- and variant-wise QC was applied in an iterative manner by using the Hail framework (https://github.com/hail-is/hail) v0.1 and the resulting high-quality WGS data for 3775 individuals were phased with Eagle 2.3.5, as described above.

In post-imputation QC, variants within+-100kB of the *UST* locus were selected, and variants with an imputation INFO (information) score < 0.3 or a minor allele frequency < 0.01 were removed.

### Covariates

In the survival analyses of the FINRISK Study, the basic model was adjusted for sex, educational classes (sex-specific and birth-cohort specific tertiles of schooling years), and the FINRISK Study survey year. The follow-up time (> 16 years/ < 16 years) and age at the end of follow-up (5 years time slots) were accounted for in the Lexis data frame in the Poisson model. In the fully adjusted model, we added covariates of systolic blood pressure (average of one to three measurements), body mass index (BMI), total cholesterol, smoking (non-/current/ex-smoker), physical activity in three classes (combined occupational, leisure and commuting activity as described in^39,40^), and diabetes.

Work-related stress, as well as insomnia, were used as covariates in the secondary analyses. One question assessed work-related stress in the FINRISK Study surveys of 1992–2002: “*How often are you troubled by having to stretch your strength to the extreme to be able to cope with your present work or workload?”.* The response options were: 1 = *almost all the time*, 2 = *quite often*, 3 = *sometimes*, 4 = *seldom*, 5 = *never*, 6 = *I do not work*. We grouped answers 1–2 (high stress) and 3–5 (low stress) together and reversed the scale. Answer 6, *I do not work*, was excluded. Insomnia was assessed in the FINRISK Study surveys of 1992–2007 with the question: “*Do you suffer from insomnia?”:* 1 = *often*, 2 = *sometimes,* 3 = *not at all.*

### Statistical analyses

We considered death and incidence of dementia as competing risks and fitted two alternative models: Poisson Cause-specific hazard model and Fine-Gray sub-distribution hazard model^31^. In multiplicative Poisson models, death or incidence of disease was modelled using the log-link function and log length of follow-up time as an offset. Incidence rate ratios (IRRs) of that model are very similar to the hazard rates (HRs) of Cox’s proportional hazards model, but without the proportionality assumption. The Poisson cause-specific hazard model has been suggested to emphasise aetiological risk factors^41^, while the Fine and Gray sub-distribution model emphasises prognostic risk.

For the Poisson model, we used Lexis data frame to include two timescales in the analysis: age and the time since start of follow-up. Cut points for age were 65, 70, 75, 80, and 85 years, and for follow-up time 16 years. R software (version 3.6.0, R Foundation for Statistical Computing, Vienna, Austria. https://www.R-project.org/.) package Epi (version 2.40) was used for the Lexis data frame and packages cmprsk (version 2.2-9) and survival (version 2.44-1.1) for survival analyses.

In the secondary analyses the Poisson model analyses were additionally adjusted for insomnia, work-related stress or the top three principal components of GWAS data (C1–C3).

All the methods were carried out in accordance with relevant guidelines and regulations.

### Fine mapping

To fine map the associations of the genetic area of rs13219957, we first examined its haplotype structure in the FINRISK Study surveys using the imputed GWAS data. Haplotypes were defined using the confidence interval algorithm in the Haploview programme^42^. In the haplotype block area, we selected all the genetic variants in linkage disequilibrium (LD), r^2^ > 0.20, with rs13219957 using PLINK (version 1.90b4.1)^43^. Those variants were studied against all-cause dementia and AD in the FINRISK Study surveys using fully adjusted Poisson model.

### Neuropathological analyses

Rs13219957 and the fine mapped variants with significant associations with all-cause dementia in the FINRISK Study surveys were studied further with neuropathological and clinical measures in the Vantaa 85+ using linear or logistic regression analyses with sex as a covariate.

### Analyses of DNA methylation analyses, *UST* expression, and open chromatin region

Methylation analyses were performed in the DILGOM subset of the FINRISK Study survey of 2007 (n = 454 with non-missing covariates) using the HumanMethylation450K BeadChip (Illumina, Inc., San Diego, CA, USA). The preprocessing, QC, and of the data were performed using the R package *minfi*^44^*.* After the background correction, methylation values were normalised using the subset quantile normalisation method (SWAN)^45^. After the standard QC procedures, we removed the following probes: (a) probes with detection *P* value > 0.01; (b) cross-reactive probes and probes containing a single nucleotide polymorphism (SNP), as described in^46^. We extracted all the available methylation sites in the *UST* gene area and untranslated regions (UTRs), which numbered 92.

The proportions of white blood cells were calculated by the Houseman method^47^. For the subsequent association analyses, we used M-values (logit-transformed beta values). For testing the associations of the genetic variants with methylation M-values of the CpGs, we used a multiple linear regression model adjusted for age, sex, smoking status, alcohol consumption, cell counts, and technical variables array and slide. The *P* values were adjusted to control the false discovery rate (FDR) in multiple hypothesis testing using the Benjamini–Hochberg procedure, and the FDR-adjusted *P* value < 0.05 was used to assess statistical significance.

Analyses examining the associations between *UST* locus SNPs and *UST* brain expression (eQTL) were conducted using publicly available data from the ROSMAP project^19^ (dorsolateral prefrontal cortex) and the GTEx project portal^20^ (the corresponding tissue, brain cortex) as well as the eGWAS Mayo project (temporal cortex)^21^ including patients with definitive AD, and non-AD individuals (mostly with other brain pathologies)^17^.

Additionally, in a dataset from the study on human microglial cells by Kosoy et al.^22^, we explored microglial cell eQTLs and the association of the *UST* locus SNPs with the open chromatin region (OCR) in the *UST* promoter region (Peak_174460) assessed using ATACseq method.

### Supplementary Information

Supplementary Information.

## Data Availability

The National FINRISK study data that supports the findings of this study are available from the THL Biobank on application https://thl.fi/en/web/thl-biobank/for-researchers/application-process.
