# Supplementary material for: Job-related exhaustion risk variant in UST is associated with dementia and DNA methylation
Source: Sci Rep. 2024 Jun 13;14:13668. doi: 10.1038/s41598-024-62600-3 (PMC11176189; doi:10.1038/s41598-024-62600-3)
Supplement: Supplementary file 1 — Supplementary Information. [file 41598_2024_62600_MOESM1_ESM.docx]

**Supplementary Figure S1.** Distribution of BRAAK stage and rs13219957 A allele carriership in the Vantaa 85+ sample. NFT, neurofibrillary tangle.

**
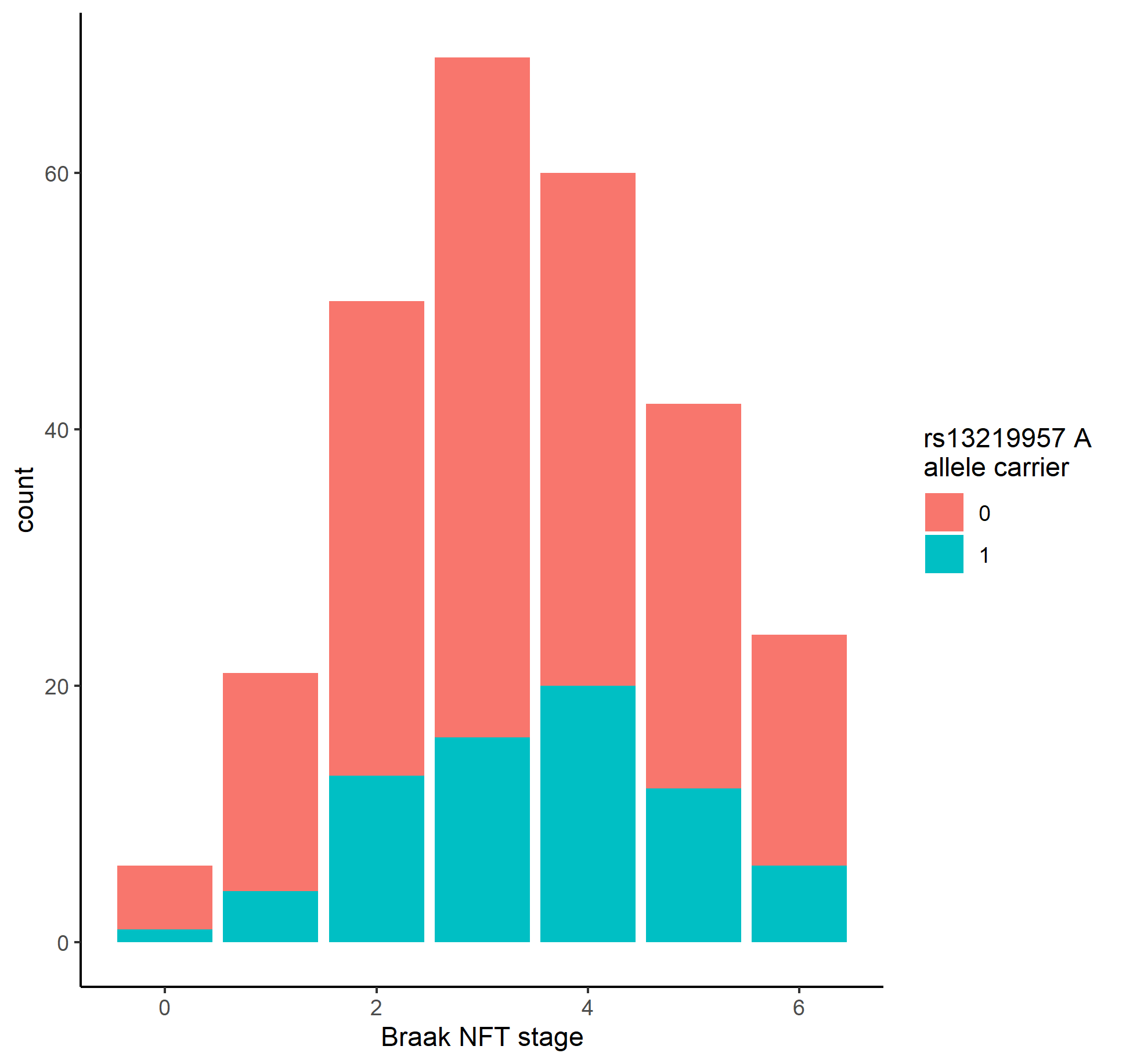
**

**Supplementary Figure S2.** Distribution of CERAD score and rs13219957 A allele carriership in the Vantaa 85+ sample. CERAD, Consortium to Establish a Registry for Alzheimer’s disease.

**
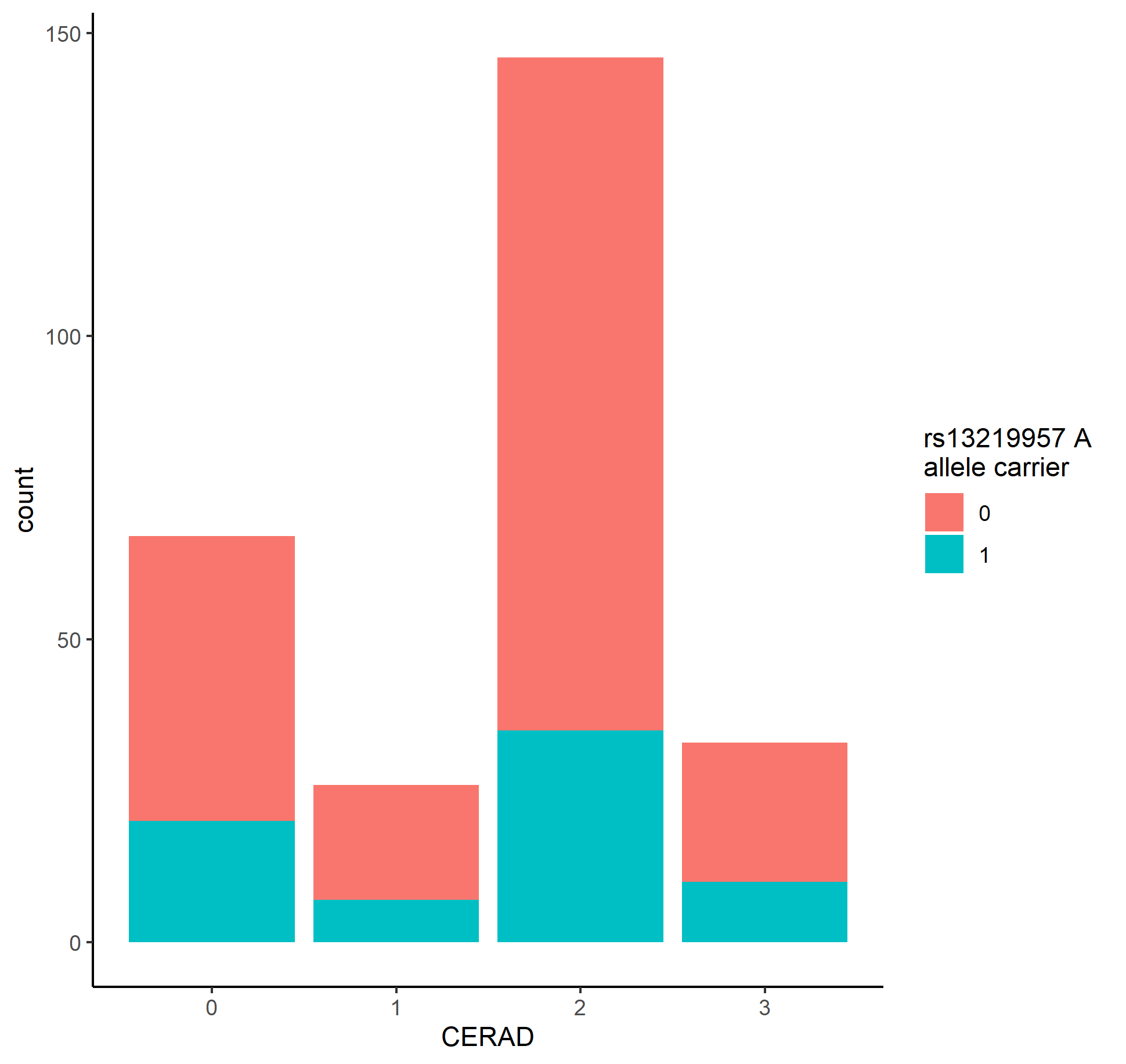
**

**Supplementary Figure S3.** Diagnosis of clinical Alzheimer’s disease and rs13219957 A allele carriership in the Vantaa 85+ sample.

**
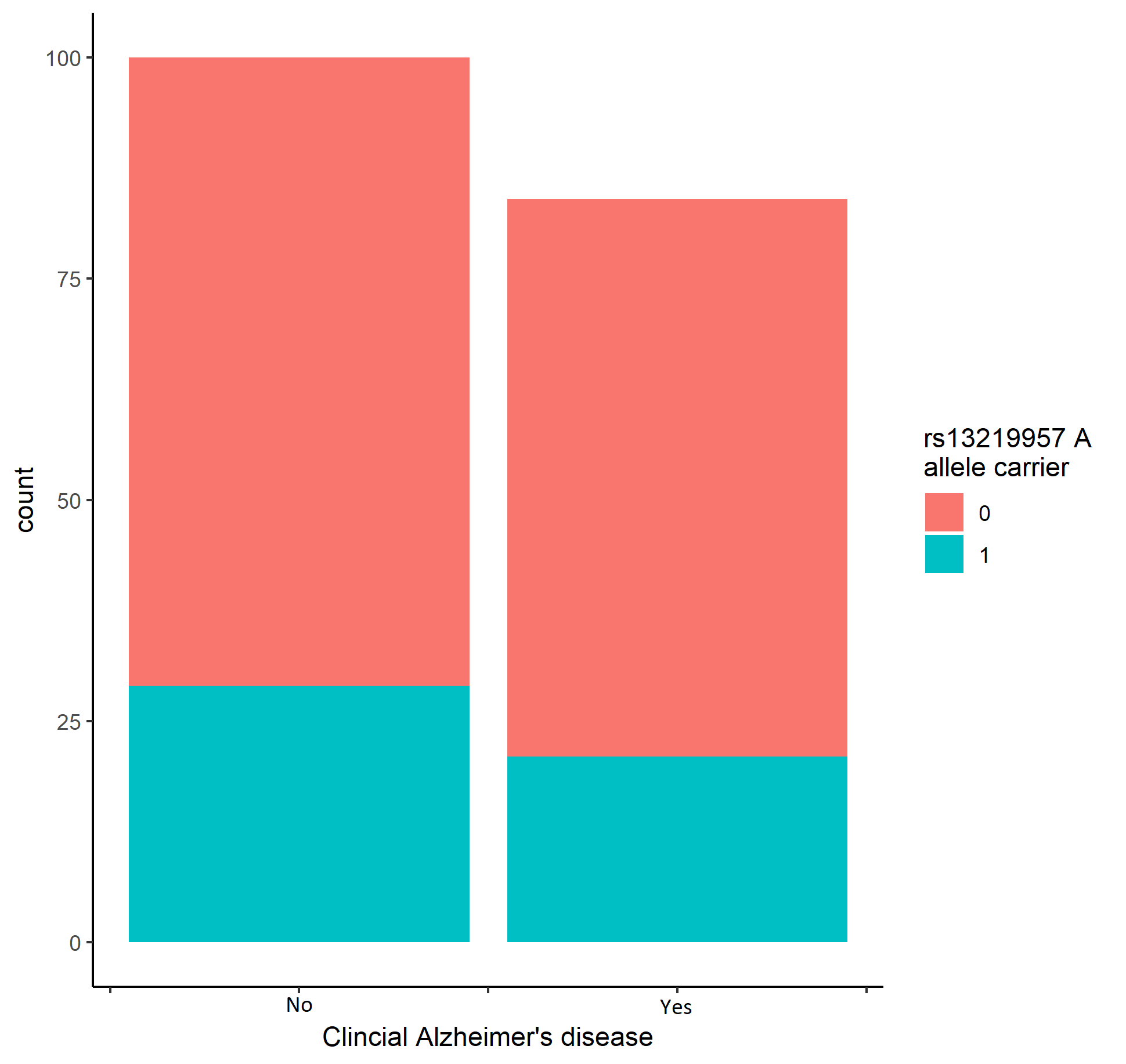
**

**Supplementary Figure S4.** Scatter plot for association of rs13219957 genotype with cg23982392 methylation status. Data points labeled for the number of minor alleles for rs75776863.

**Supplementary Table S1.** Baseline characteristics and dementia and mortality of the study population according to genotyping status.

| **Trait** | **rs13219957**  **genotype available (N total=26 693)** | **rs3219957**  **genotype**  **missing (N total=2710)** | **Test of difference P value** |
| --- | --- | --- | --- |
| FINRISK 1992 (%) | 5594/6051 (92.4) | 457/6051 (7.6) |  |
| FINRISK 1997(%) | 6874/8446 (58.0) | 1572/8446 (18.6) |  |
| FINRISK 2002(%) | 8239/8648 (95.3) | 409/8648 (4.7) |  |
| FINRISK 2007(%) | 5986/6258 (95.7) | 272/6258 (4.3) | <0.001^b^ |
| Female sex, (%) | 14 254/26 693 (53.4) | 1104/2710 (40.7) | <0.001^b^ |
| Baseline age, mean (SD) | 47.7 (13.1) | 50.8(13.8) | <0.001^a^ |
| All-cause dementia, (%) | 1540/26 693 (5.8) | 262/2710 (9.7) | <0.001^b^ |
| Age of onset for all-cause dementia, mean | 76.1 (7.5) | 76.9 (7.7) | 0.097 |
| Deaths, (%) | 3824/26 693 (14.3) | 783/2710 (28.9) | <0.001^b^ |
| Death age, mean (SD), y | 71.5 (11.2) | 73.5 (10.1) | <0.001^a^ |
| Years of education, mean (SD) | 11.9(3.9) | 11.3(4.2) | <0.001^a^ |
| Exhaustion  often, (%) | 2926/26267 (11.1) | 284/2639 (10.8) | <0.001^b^ |
| Insomnia  often, (%) | 2233/26 320 (8.4) | 222/2652 (8.5) | >0.05 |
| Physical activity low, (%) | 2095/25 313 (8.3) | 233/2408 (9.7) |  |
| Physical activity moderate, (%) | 9108/25 313 (36.0) | 954/2408 (39.6) |  |
| Physical activity high, (%) | 14 119/25 313 (55.7) | 1221/2408 (50.7) | <0.001^b^ |
| BMI, mean (SD), kg/m^2^ | 26.7(4.7) | 26.9 (4.6) | 0.030 ^a^ |
| Systolic blood pressure, mean (SD), mmHG | 134.8 (19.7) | 137.6 (20.9) | <0.001^a^ |

SD, standard deviation; BMI, body mass index.  ^a^One-way ANOVA, equal variances and means. ^b^Pearson Chi-Square test.

**Supplementary Table S2.** Association of rs13219957 with all-cause dementia in the fully adjusted Poisson cause-specific hazard model with additional covariate of GWAS population components C1–C3, and normal fully adjusted Poisson cause-specific hazard model with the same sample.

|  |  | **Fully-adjusted model + C1-C3** | **Fully-adjusted model in the sample with GWAS data** |
| --- | --- | --- | --- |
| **All-cause dementia** | N  (cases) | **Poisson Cause-specific hazard model***  **IRR (95%)** | **Poisson Cause-specific hazard model****  **IRR (95%)** |
| **rs13219957**  GG | 13 588 (753) | 1.00 | 1.00 |
| GA | 4633 (253) | 1.02(0.88-1.17) | 1.01(0.88-1.17) |
| AA | 389 (28) | 1.26 (0.86-1.84) | 1.25(0.86-1.83) |

*Model adjusted for population components C1-C3, FINRISK survey year, sex, educational class, body mass index, systolic blood pressure, total cholesterol, smoking, physical activity, and diabetes. Follow-up time and age at the end of the follow-up were accounted for in the Lexis data frame. **Model adjusted for FINRISK survey year, sex, educational class, body mass index, systolic blood pressure, total cholesterol, smoking, physical activity, and diabetes. Follow-up time and age at the end of the follow-up were accounted for in the Lexis data frame. IRR, Incidence Rate Ratio.

**Supplementary Table S3.** Associations of rs13219957 or missing genotype data with incident all-cause dementia, Alzheimer’s disease (AD), non-AD dementia, and the competing risk of death in basic cause-specific hazard model (Poisson).

|  |  | **Cause-specific hazard model (Poisson)** |  |
| --- | --- | --- | --- |
| **Trait** | **N Basic Model (cases)** | **Basic model***  **IRR (CI 95%)** |  |
| **All-cause dementia** |  |  |  |
| **rs13219957**  GG | 19 223 (1062) | 1 (reference) |  |
| GA | 6565 (362) | 0.99 (0.88-1.12) |  |
| AA | 546 (42) | 1.37 (1.00-1.86) |  |
| NA | 2560 (222) | 1.11 (0.95-1.29) |  |
| **Alzheimer’s disease** |  |  |  |
| **rs13219957**  GG | 19 223 (877) | 1 (reference) |  |
| GA | 6565 (294) | 0.98 (0.86-1.11) |  |
| AA | 546 (29) | 1.14 (0.79-1.66) |  |
| NA | 2560 (178) | 1.12 (0.95-1.32) |  |
| **Non-AD dementia** |  |  |  |
| **rs13219957**  GG | 19 223 (185) | 1 (reference) |  |
| GA | 6565 (68) | 1.04 (0.82-1.43) |  |
| AA | 546 (13) | 2.29 (1.31-4.02) |  |
| NA | 2560 (44) | 1.09 (0.77-1.52) |  |
| **Competing risk of death**  **rs13219957**  GG | 19223 (2196) | 1 (reference) |  |
| GA | 6565 (778) | 1.05 (0.99-1.12) |  |
| AA | 546 (52) | 0.81 (0.67-0.994) |  |
| NA | 2560 (533) | 1.28 (1.19-1.37) |  |

Basic model adjusted for follow-up time (10-year time slots), age at the end of follow-up (5-year time slots), FINRISK survey year, sex, and educational class. CI, confidence interval; IRR, incidence rate ratio.

**Supplementary Table S4.** Hardy-Weinberg Equilibrium (HWE) for rs13219957 in the FINRISK study sample (N=26 693).

| **Genotype** | **Observed genotype frequencies** | **Idealized genotype frequencies based on HWE** |
| --- | --- | --- |
| GG | 0.730 | 0.731 |
| GA | 0.249 | 0.248 |
| AA | 0.021 | 0.021 |

HWE, Hardy-Weinberg Equilibrium

**Supplementary Table S5.** Association of rs13219957 with all-cause dementia in the fully adjusted Poisson cause-specific hazard model with additional covariates of insomnia, work stress, and depressive mood.

|  | **Fully adjusted model + insomnia** | | **Fully adjusted model + work-related stress** | | **Fully adjusted model + depressive mood** | |  |
| --- | --- | --- | --- | --- | --- | --- | --- |
| **All-cause dementia** | N  (cases) | **Poisson cause-specific hazard model***  **IRR (95%)** | N  (cases) | **Poisson cause-specific hazard model****  **IRR (95%)** | N  (cases) | **Poisson cause-specific hazard model****  **IRR (95%)** |  |
| rs13219957  GG | 16 936 (894) | 1.00 | 9820 (270) | 1.00 | 14 027 (808) | 1.00 |  |
| GA | 5806 (309) | 1.01(0.88-1.15) | 3339 (93) | 1.05(0.83-1.33) | 4745 (281) | 1.04 (0.90-1.19) |  |
| AA | 492 (39) | 1.39(1.01-1.91) | 273 (16) | 1.80(1.08-2.98) | 405 (37) | 1.47 (1.06-2.05) |  |

*Model adjusted for insomnia, FINRISK survey year, sex, educational class, body mass index, systolic blood pressure, total cholesterol, smoking, physical activity, and diabetes. Follow-up time and age at the end of the follow-up were accounted for in the Lexis data frame. **Model adjusted for work-related stress, FINRISK survey year, sex, educational class, body mass index, systolic blood pressure, total cholesterol, smoking, physical activity, and diabetes. Follow-up time and age at the end of the follow-up were accounted for in the Lexis data frame. Includes FINRISK surveys 1992, 1997, and 2002. IRR, Incidence Rate Ratio.

**Supplementary Table S6.** Baseline characteristics and dementia and mortality of the study population according to status of methylation data.

| **Trait** | **Methylation data available (N total=511)** | **Methylation data unavailable (N total=5747)** | **Test of difference P value** |
| --- | --- | --- | --- |
| Female sex, (%) | 274/511 (53.6) | 3050/5747 (53.1) | 0.81^b^ |
| Baseline age, mean (SD) | 51.6 (13.8) | 50.3 (14.0) | 0.047 ^a^ |
| All-cause dementia, (%) | 18/511 (3.5 %) | 172/5747 (3.0 %) | 0.50 ^b^ |
| Age of onset for all-cause dementia, mean | 74.6 (6.0) | 75.6 (4.2) | 0.47 ^a^ |
| Deaths, (%) | 37/511 (7.2) | 367/5747 (6.4) | 0.45 ^b^ |
| Death age, mean (SD), y | 71.5 (7.6) | 69.9 (9.7) | 0.33 ^a^ |
| Years of education, mean (SD) | 13.7 (3.9) | 12.7 (4.0) | P<0.001 |
| Exhaustion  often, (%) | 66/503 (13.1) | 813/5530 (14.7) | 0.19 ^b^ |
| Insomnia  often, (%) | 55/504 (10.9) | 522/5542 (9.4) | 0.52 ^b^ |
| Physical activity low, (%) | 25/502 (5.0) | 431/5464 (7.9) |  |
| Physical activity moderate, (%) | 189/502 (37.6) | 2010/5464 (36.8) |  |
| Physical activity high, (%) | 288/502 (57.4) | 3023/5464 (55.3) | 0.063 ^b^ |
| BMI, mean (SD), kg/m^2^ | 26.8 (4.7) | 27.13 (4.9) | 0.18 ^a^ |
| Systolic blood pressure, mean (SD), mmHG | 134.1 (19.9) | 134.6 (19.9) | 0.61 ^a^ |

SD, standard deviation; BMI, body mass index.  ^a^One-way ANOVA, equal variances and means. ^b^Pearson Chi-Square test.

**Supplementary Table S7.** Association of rs75776863 with incident all-cause dementia in the Poisson cause-specific hazard model.

|  |  |  | **Poisson Cause-specific hazard model** | |
| --- | --- | --- | --- | --- |
| **Trait** | **N basic Model (cases)** | **N fully adjusted model (cases)** | **Basic model***  **IRR (CI 95%)** | **Fully adjusted model****  **IRR (CI 95%)** |
| rs75776863  CC | 22 652 (1267) | 20 191 (1067) | 1.00 | 1.00 |
| CT | 2221 (155) | 2009 (130) | 1.22(1.03-1.44) | 1.17(0.98-1.41) |
| TT | 52 (0) | 46 (0) | NA | NA |

*Model adjusted for FINRISK survey year, sex, educational class **Model adjusted for FINRISK survey year, sex, educational class, body mass index, systolic blood pressure, total cholesterol, smoking, physical activity, and diabetes. Follow-up time and age at the end of the follow-up were accounted for in the Lexis data frame. NA, not analysed because of low number of TT homozygotes.

**Supplementary Table S8.** Association of rs13219957 with incident all-cause dementia in the Poisson cause-specific hazard model in the sample with GWAS data. Homozygotes and heterozygotes for the minor allele of rs75776863 excluded.

|  |  |  | **Poisson Cause-specific hazard model** | |
| --- | --- | --- | --- | --- |
| **Trait** | **N basic Model (cases)** | **N fully adjusted model (cases)** | **Basic model***  **IRR (CI 95%)** | **Fully adjusted model****  **IRR (CI 95%)** |
| rs13219957  GG | 16 673 (917) | 14 854 (776) | 1.00 | 1.00 |
| GA | 4701 (242) | 4185 (201) | 0.91 (0.79-1.05) | 0.93 (0.79-1.08) |
| AA | 319 (23) | 290 (21) | 1.44 (0.95-2.17) | 1.54 (0.996-2.37) |

*Model adjusted for FINRISK survey year, sex, educational class **Model adjusted for FINRISK survey year, sex, educational class, body mass index, systolic blood pressure, total cholesterol, smoking, physical activity, and diabetes. Follow-up time and age at the end of the follow-up were accounted for in the Lexis data frame. NA, not analysed because of low number of TT homozygotes.

**Supplementary Table S9.** Association of *UST* locus SNPs with cortical brain RNA expression of *UST* in the ROSMAP study ^1^ in the GTEx (Genotype-Tissue-Expression) Portal^2^, and in the eGWAS Mayo data^3^.

| ROSMAP, N=534 | | | | | GTEx, N=205 | | eGWAS Mayo AD, N=194 | | | eGWAS Mayo non-AD, N=186 | | |
| --- | --- | --- | --- | --- | --- | --- | --- | --- | --- | --- | --- | --- |
| SNP | A1 | Beta | SE | P | NES | P | BETA | SE | P | BETA | SE | P |
| rs6570912 | C | -0.050 | 0.018 | 0.0051 | -0.14 | 0.042 | -0.051 | -1.958 | 0.052 | -0.023 | -0.94 | 0.35 |
| rs992811 | G | -0.051 | 0.018 | 0.0042 | -0.15 | 0.030 | NA | NA | NA | NA | NA | NA |
| rs9322161 | T | -0.048 | 0.018 | 0.0076 | -0.15 | 0.030 | -0.051 | -1.955 | 0.052 | -0.023 | -0.94 | 0.35 |
| rs6936210 | C | -0.048 | 0.018 | 0.0076 | -0.14 | 0.037 | NA | NA | NA | NA | NA | NA |
| rs7769776 | G | -0.049 | 0.018 | 0.0064 | -0.15 | 0.030 | -0.051 | -1.955 | 0.052 | -0.023 | -0.94 | 0.35 |
| rs1482393 | T | -0.049 | 0.018 | 0.0068 | -0.15 | 0.030 | -0.051 | -1.955 | 0.052 | -0.023 | -0.94 | 0.35 |
| rs6570913 | A | -0.048 | 0.018 | 0.0071 | -0.15 | 0.030 | -0.051 | -1.955 | 0.052 | -0.023 | -0.94 | 0.35 |
| rs6941730 | T | -0.049 | 0.018 | 0.0070 | -0.15 | 0.030 | -0.051 | -1.955 | 0.052 | -0.023 | -0.94 | 0.35 |
| rs11155608 | A | -0.049 | 0.018 | 0.0062 | -0.15 | 0.030 | NA | NA | NA | NA | NA | NA |
| rs13219957 | A | -0.040 | 0.026 | 0.12 | -0.079 | 0.41 | 0.0089 | 0.1947 | 0.8459 | -0.028 | -0.62 | 0.54 |
| A1, Minor allele; SE, Standard error; NES, normalized effect size; NA, not available; AD, Alzheimer’s disease. | | | | | | | | | | | | |

**Supplementary Table S10.** Association of the *UST* locus SNPs with microglial RNA expression of *UST* in the Kosoy et al. study ^4^.

| SNP | A1 | Beta | SE | Z score random | P |
| --- | --- | --- | --- | --- | --- |
| rs6570912 | C | 0.0081 | 0.072 | 0.154 | 0.88 |
| rs992811 | G | 0.0081 | 0.073 | 0.148 | 0.88 |
| rs9322161 | T | 0.013 | 0.073 | 0.286 | 0.77 |
| rs6936210 | C | 0.014 | 0.073 | 0.285 | 0.78 |
| rs7769776 | G | 0.015 | 0.073 | 0.235 | 0.81 |
| rs1482393 | T | 0.016 | 0.073 | 0.236 | 0.81 |
| rs6570913 | A | 0.013 | 0.073 | 0.286 | 0.77 |
| rs6941730 | T | 0.014 | 0.073 | 0.285 | 0.78 |
| rs11155608 | A | 0.014 | 0.073 | 0.285 | 0.78 |
| rs13219957 | A | -0.066 | 0.10 | 0.640 | 0.52 |

A1, Minor allele; SE, Standard error

**Supplementary Table S11.** Association of the *UST* locus SNPs with the open chromatin region (OCR) in the *UST* promoter region. ATACseq data by Kosoy et al ^4^.

| SNP | A1 | Beta | Z score random | P |
| --- | --- | --- | --- | --- |
| rs6570912 | C | -0.15 | 1.935 | 0.053 |
| rs992811 | G | -0.15 | 1.935 | 0.053 |
| rs9322161 | T | -0.14 | 1.918 | 0.055 |
| rs6936210 | C | -0.14 | 1.918 | 0.055 |
| rs7769776 | G | -0.17 | 2.144 | 0.032 |
| rs1482393 | T | -0.17 | 2.144 | 0.032 |
| rs6570913 | A | -0.14 | 1.918 | 0.055 |
| rs6941730 | T | -0.14 | 1.918 | 0.055 |
| rs11155608 | A | -0.14 | 1.918 | 0.055 |
| rs13219957 | A | -0.096 | 0.852 | 0.394 |

SNP, single nucleotide polymorphism; A1, minor allele.

**References for Supplementary Material**

1. Ng, B. *et al.* An xQTL map integrates the genetic architecture of the human brain’s transcriptome and epigenome. *Nat Neurosci* **20**, 1418–1426 (2017).

2. Carithers, L. J. *et al.* A Novel Approach to High-Quality Postmortem Tissue Procurement: The GTEx Project. *Biopreservation and Biobanking* **13**, 311–319 (2015).

3. Zou, F. *et al.* Brain expression genome-wide association study (eGWAS) identifies human disease-associated variants. *PLoS Genet* **8**, e1002707 (2012).

4. Kosoy, R. *et al.* Genetics of the human microglia regulome refines Alzheimer’s disease risk loci. *Nat Genet* **54**, 1145–1154 (2022).
